# Identification of Proteins Differentially Expressed in the Striatum by Melatonin in a Middle Cerebral Artery Occlusion Rat Model—a Proteomic and *in silico* Approach

**DOI:** 10.3389/fnins.2018.00888

**Published:** 2018-12-10

**Authors:** Fawad Ali Shah, Amir Zeb, Tahir Ali, Tahir Muhammad, Muhammad Faheem, Sayed Ibrar Alam, Kamran Saeed, Phil-Ok Koh, Keun Woo Lee, Myeong Ok Kim

**Affiliations:** ^1^Division of Applied Life Science (BK 21), College of Natural Science, Gyeongsang National University, Jinju, South Korea; ^2^Department of Pharmacology, Riphah Institute of Pharmaceutical Sciences, Riphah International University Islamabad, Rawalpindi, Pakistan; ^3^Department of Pharmacy, Faculty of Life Science, Sarhad University of Science and Information Technology, Peshawar, Pakistan; ^4^Department of Anatomy, College of Veterinary Medicine, Research Institute of Life Science, Gyeongsang National University, Jinju, South Korea

**Keywords:** melatonin, striatum, ischemic stroke, docking, neuroprotection

## Abstract

Ischemic stroke is characterized by permanent or transient obstruction of blood flow, which initiates a cascading pathological process, starting from acute ATP loss to subsequent membrane depolarization, glutamate excitotoxicity, and calcium overload. Melatonin is a potent antioxidant that exerts protective effects in different experimental stroke models. In this study, melatonin effects were demonstrated by a proteomic and *in silico* approach. The proteomic study identified differentially expressed proteins by 2D gel electrophoresis in the striatum 24 h after middle cerebral artery occlusion. Proteomic analysis revealed several proteins with aberrant expression and was validated by western blot and immunofluorescence analysis. Homology modeling was performed to build 3D structures for γ-enolase, thioredoxin (TRX), and heat shock 60 (HSP60) by the template crystal structures using a protein data bank as a sequence database. The structure refinement of each model was achieved by energy minimization via molecular dynamic simulation, and the generated models were further assessed for stability by Procheck and ProSA. The models were processed for docking analysis using AutoDock Vina, and post-docking analysis was determined by discovery studio. The proteomic study showed decreased expression of γ-enolase, TRX, and protein phosphatase 2A subunit B and increased expression of collapsin response mediator protein 2 and HSP60 in the striatum after ischemic injury. Treatment with melatonin modulated the expression profiles of these proteins. This study demonstrated the neuroprotective role of melatonin in the ischemic striatum using a proteomic and *in silico* approach. Collectively, melatonin may act in a multimechanistic way by modulating the expression of several proteins in the ischemic striatum.

## Introduction

Ischemic stroke is the most frequent cause of mortality depending upon race and demographic location (Mozaffarian et al., 2015). Stroke is also the significant cause of human sufferings, and the tissue plasminogen activator is the only drug approved for reversal of stroke torment by recanalizing the obstructed vessel (vascular strategy for combating stroke). A consensus based on experimental results recommends that recanalization (vascular therapy) is not sufficient to attenuate ischemic damage; however, a neuroprotective strategy is more productive, and it can impede the progression of ischemic lesion, counteract many biochemical steps of ischemic cascade, and provide an appropriate therapeutic choice. Therefore, the potential neuroprotective effects of melatonin were determined using a proteomic approach in this study.

Among various endogenous and synthetic neuroprotective compounds (including estrogens and progesterone), melatonin is the most extensively studied neuroprotective compound. As a natural indole hormone produced by the pineal gland and many other tissues such as retina, gut, and glial cells in mammals, melatonin is an effective antioxidant, besides a regulator of circadian and circannual cycles through G protein-coupled receptors, i.e., melatonin type 1 and 2 receptors (Venegas et al., 2012; Vriend and Reiter, 2015; Lacoste et al., 2015). Melatonin readily crosses the blood-brain barrier (BBB) because of its amphiphilic character, and melatonin receptors are widely distributed in the central nervous system (Lacoste et al., 2015). Moreover, melatonin has a broad interacting profile with intracellular proteins, including quinone reductase 2 (melatonin type 3 receptor) (Tan et al., 2007). Therefore, the biological activities of melatonin cannot be attributed to a single pathway or receptor but involve many targets including transduction pathways. Thus, receptor-dependent and independent actions, the low toxicity profile, and excellent clinical safety records make melatonin an ideal candidate as a neuroprotective agent (Ramos et al., 2017).

The neuroprotective effects of melatonin on ischemic stroke have been extensively studied in *in vitro* and *in vivo* models. The protection of melatonin against ischemic cell death could be attributed to a variety of cellular and molecular mechanisms, including its antioxidant and anti-inflammatory activities (Mauriz et al., 2013; García et al., 2014). A high concentration of melatonin directly eradicates free radicals, but a relatively low concentration of melatonin activates antioxidant enzymes (Rodriguez et al., 2004). Several signaling pathways, including the pro-survival phosphor-inositol 3 kinase (PI3K)/ protein kinase B and mitogen-activated protein kinases (MAPK) and oxidative stress-related nuclear factor (erythroid-derived 2)-like 2 (NRF2), sirtuin 1, and endothelin-1, might be involved in the role of melatonin in brain ischemia (Andrabi et al., 2015). Many studies have demonstrated that melatonin counteracts the deleterious effects of ischemic stroke in animal models by promoting BBB integrity and neurogenesis (Lee et al., 2014; Alluri et al., 2016). Furthermore, melatonin diminishes the infarct volume, reduces brain water content, and improves neurologic scores in focal cerebral ischemia.

The extent of neuronal injury triggered by middle cerebral artery occlusion (MCAO) depends upon the duration of occlusion (Fluri et al., 2015). Permanrent MCAO induces the most uniform infarction that frequently involves the neocortex and striatum. Comparatively, blood flow is lower to the striatum than to the cortex. The striatum is supplied by tiny unidirectional vessels from MCA, and there is no collateral connection to this subcortical area from the surrounding vasculatures (Fluri et al., 2015). Thus, MCA occlusion completely cuts off blood supply to this important region in the brain, and the striatum may be severely hit by ischemic stroke.

*In silico* and proteomic studies help understand the biochemical mechanism and thus can unknot the complex signaling network, which controls cellular function including cell survival and death. This study aimed to delineate significant targets of melatonin in the ischemic striatum. We hypothesized that melatonin modulates the expression of proteins in the striatum and may thus potentially ameliorate the molecular and organ/tissue damage associated with ischemic stroke.

## Materials and Methods

### Animals and Drug Treatment

Male SD rats (weight, 230–250 g; age, 7–9 weeks) (n = 100) were used in this study, and they were obtained from the local breeding facility at Gyeongsang National University. The experimental procedures were carried out according to the protocol approved by the animal ethics committee (Approval ID: 125-IACUC) of Gyeongsang National University, Republic of Korea. The rats were divided into 4 groups. We were not blinded to the allocation of rats; instead, we randomly divided these rats into the following groups according to the criteria that the similar weight animals are kept in the same group under the same experimental condition: (1) Vehicle-treated control rats (Sham); (2) Middle cerebral artery occlusion rats (MCAO); (3) Melatonin-treated rats undergoing MCAO (Mela + MCAO); 4. Melatonin-treated sham rats (Mela+Sham).

A single dose of melatonin (Sigma, St. Louis, MO, United States) (5 mg/kg) or vehicle was administered intraperitoneally 30 min before ischemia. This dose of melatonin has shown maximum neuroprotective effects in focal cerebral ischemia based on pharmacokinetic and dose-response studies (Lee et al., 2005). In total, 10 rats died during experimental procedures, including 6 from the MCAO group, 3 from the Mela + MCAO group, and 1 from the sham-operated group.

### Middle Cerebral Artery Occlusion Surgery

Middle cerebral artery occlusion was operated using a previously described method (Shah et al., 2016; Park et al., 2018). Briefly, all main arteries involved in blood occlusion were exposed, including the common carotid artery, external carotid artery, and internal carotid artery. The thinner occipital artery and superior thyroid artery (originated from the external carotid) were ligated with 6-0 silk sutures. The external carotid artery was knotted with 6-0 silk sutures, and a nylon filament with a blunt rounded tip of about 30 mm in length was inserted into the internal carotid artery versus the external carotid artery. The filament was advanced until a resistance was felt, demonstrating that the middle cerebral artery was occluded. The sham-operated animals were subjected to the same procedures except for the filament insertion. At 24 h after onset of permanent occlusion, animals were decapitated, and brain tissues were collected.

### Proteomics of the Striatal Tissues

Proteomic analysis was carried out using a previously described method (Shah et al., 2014). Briefly, the right striatum was isolated from all experimental groups and was homogenized in a buffer solution (8 M urea, 4% 3-[(3-Cholamidopropyl) dimethylammonio]-1-propanesulfonate hydrate [CHAPS], ampholytes, and 40 mM Tris–HCl), followed by centrifugation. The resulted supernatant was discarded; the pallet was dissolved in the lysis buffer, and the protein concentration was determined by Bradford method (Bio-Rad, Hercules, CA, United States) according to the manufacturer’s protocol. Immobilized pH gradient (IPG) gel strips (range pH 4–7 and 6–9, Bio-Rad) were incubated in the rehydration buffer (8 M urea, 2% CHAPS, 20 mM dithiothreitol [DTT], 0.5% IPG buffer, and bromophenol blue) for 13 h at room temperature. The assayed protein samples were loaded on IPG strips (pH 4-7 and 6-9) via the sample cup and proceed for first dimension isoelectric focusing (IEF) using Ettan IPGphor 3 (GE Healthcare, Bio-Rad) with the following protocol: l,250 V (15 min), 10,000 V (3 h), and then 10,000–50,000 V. At end of the first dimension IEF, the strips were incubated in the equilibration buffer (6 M urea, 30% glycerol, 2% sodium dodecyl sulfate [SDS], 50 mM Tris-HCl, and bromophenol blue) containing DTT and iodoacetamide. The strips were then loaded onto gradient gels (7.5–17.5%), and the second-dimension electrophoresis was performed on a Protein-II XI electrophoresis equipment (Bio-Rad) at 5 mA per gel for 2 h, followed by 10 mA per gel at 10°C until the bromophenol blue dye migrated off the bottom of the gel. The steps used for staining the gel included fixation (12% acetic acid, 50% methanol), impregnation in a silver solution (0.2% silver nitrate, 0.75 ml/L formaldehyde), and developing (0.2% sodium carbonate, 0.5 ml/L formaldehyde). Gel images were acquired, and differentially expressed protein spots were excised and destained. Gel particles were digested in the trypsin-containing buffer, and the extracted peptides were analyzed using a Voyager-DETM STR biospectrometry workstation (Applied Biosystem, Forster City, CA, United States) for peptide mass fingerprinting. Database searches were carried out using MS-Fit and ProFound software. SWISS-PROT and NCBI were used as protein sequence databases.

### Western Blot

For western blot analysis, samples were homogenized in the lysis buffer (1 M Tris–HCI, 5 M sodium chloride, 0.5% sodium deoxycholate, 10% sodium dodecyl sulfate, 1% sodium azide, and 10% NP-40) with phenylmethylsulfonyl fluoride as protein inhibitor. The homogenate was sonicated and centrifuged, and protein concentration was then determined by Bicinchoninic Acid kit (Pierce, Rockford, IL, United States) according to the manufacturer’s guideline. An equal amount of proteins (30 μg per sample) were electrophoresed on 10% SDS-PAGE gels, followed by transferring the protein to polyvinylidene fluoride (PVDF) membranes (Millipore, Billerica, MA, United States). The PVDF membranes were blocked with skim milk at room temperature to minimize non-specific antibody binding and were then incubated with primary antibodies overnight at 4°C. Subsequently, the membranes were incubated with appropriate secondary antibodies, and protein bands were detected using ECL detection reagents according to the manufacturer’s instruction (Amersham Pharmacia Biotech, Piscataway, NJ, United States). The antibodies used included anti-γ-enolase, anti-heat shock protein 60 (HSP60), anti-thioredoxin (TRX), and anti-β-Actin from Santa Cruz Biotechnology (Santa Cruz, CA, United States) and anti-collapsin response mediator protein 2 (CRMP2) and anti-protein phosphatase 2A subunit B (PP2A) from cell signaling technology.

### Tissue Collection for Morphology Analysis

Five rats were used for morphology analysis in each group. Brain tissues were fixed in 4% paraformaldehyde and embedded in paraffin, and 4 μm coronary sections were cut using a rotary microtome. The following staining techniques were used in this study.

### Cresyl Violet Staining

Tissue sections on coated slides were de-paraffinized with three different absolute xylenes and were rehydrated with ethyl alcohol (from 100% [absolute] to 70%). The slides were rinsed with distilled water and immersed in 0.01 M phosphate-buffered saline (PBS) for 10 min. Cresyl violet acetate (0.5% [w/v]; Sigma) was dissolved in distilled water, and a few drops of glacial acetic acid were then added. Brain sections were stained with cresyl violet solution for approximately 20 min. The slides were rinsed with distilled water and then dehydrated in ethyl alcohol (70, 95, and 100%). The slides were cleared with xylene and mounted with glass coverslips. The slides were imaged with an Olympus microscope, and the images were analyzed by ImageJ, a computer-based program. In total, 5 images per slide were acquired for each group, and specifically, neuropil and neuronal size and shape were focused in these images. The TIF images were optimized to the same threshold intensity for pyknotic, red, and ghost neurons in all groups.

### Immunofluorescence Analysis

After deparaffinization, the slides were autoclaved in 0.1 M sodium citrate (pH 6) for antigen retrieval, washed with PBS, and incubated with 5% normal serum depending upon the sources of the secondary antibodies used. The slides were incubated with mouse polyclonal γ-enolase, HSP60, TRX, Ionized calcium binding adaptor molecule 1 (Iba-1), glial fibrillary acidic protein (GFAP), and 8-oxoguanine antibodies from Santa Cruz Biotechnology and the rabbit monoclonal CRMP2 antibody from cell signaling overnight at 4°C. Subsequently, after washing with PBS, the slides were incubated with fluorescent-labeled secondary antibodies (Santa Cruz Biotechnology) for signal amplification in a dark chamber and were then mounted with UltraCruz mounting medium (Santa Cruz Biotechnology). Immunofluorescence images (five images per slide) were captured using a confocal scanning microscope (Flouview FV 1000, Olympus, Japan). ImageJ software was used to quantitatively determine fluorescence intensity of the same region of the striatum/total area for all groups by optimizing background of images according to the threshold intensity and analyze the immunofluorescence intensity at the same threshold intensity for all groups. The fluorescence intensity is expressed as the relative integrated density of the samples relative to the control.

### Immunohistochemical Analysis

After antigen retrieval, the slides were incubated with 3% hydrogen peroxidase to quench endogenous peroxidase and were subsequently blocked with 5% serum depending upon the sources of secondary antibodies used. After blocking, the slides were incubated overnight with anti-PP2A (Cell signaling technology), p-c-Jun N-terminal kinase (JNK), and caspase3 (Santa Cruz Biotechnology) antibodies, followed by treatment with appropriate biotinylated secondary antibodies for 2 h and successively with ABC reagents (Standard Vectastain ABC Elite Kit; Vector Laboratories, Burlingame, CA, United States) for 1 h at room temperature. The sections were washed with PBS and stained in 3, 3′-Diaminobenzidine tetrahydrochloride solution; they were then washed with distilled water, dehydrated in graded ethanol (70, 95, and 100%), fixed in xylene, and cover-slipped by a mounting medium. Immunohistochemical results were analyzed by a light microscope (Olympus, Japan), which was connected to a digital photomicroscopy system. Immunohistochemical TIF images (five images per slide) were captured with a light microscope. ImageJ software was used to quantitatively determine hyperactivated p-JNK, PP2A, and caspase3 in the striatum/total area by optimizing background of images according to the threshold intensity and analyze p-JNK, PP2A, and Caspase3 positive cells at the same threshold intensity for all groups. The intensity is expressed as the relative integrated density of the samples relative to the control.

### Bioinformatics Resources

The amino acid sequences of target proteins (HSP60, CRMP2, γ-enolase, TRX, and PP2A) in rats were downloaded from UniProt database^1^ in FASTA format. To identify the best template structure for homology modeling, the sequence of the target protein was aligned in BLASTp (Basic Local Alignment Searching Tool for protein) using RCSB as the protein sequence database. The templates were ranked according to sequence identity, sequence coverage, and *E*-value. The topmost ranked structure was taken as the best template for the model generation of corresponding proteins.

Homology modeling of target proteins was performed by an online server of SWISS-MODEL^2^. Briefly, template sequences were fed to the automated modeling program, and models were then generated (Biasini et al., 2014). The best model was selected based on the quality estimation score and overall structure similarity. The structure refinement of these models was achieved by energy minimization via molecular dynamic (MD) simulation using GROMACSv5.0.6 with CHARMm27 force-field parameterization (Abraham et al., 2015). Briefly, for each protein, the system was prepared in a dodecahedron box, filled with the TIP3P water model. For atomic representation, the CHARMm27 force-field parameters were applied, and the system was further neutralized by adding Na^+^ and/or Cl^-^ counter ions (Zoete et al., 2011). The well-neutralized systems were subjected to energy minimization by applying the steepest descent algorithm implanted in GROMACS v5.0.6. The energy minimization parameters were optimized to 50000 steps at 10.0 kJ/mol. The energy minimization was further verified by calculating the potential energy of the system. The generated models were further assessed for stability and overall protein quality by validation tests such as Procheck^3^ and ProSA^4^. The Procheck verifies the occurrence of residues in Ramachandran plot and deals with phi and psi angles of residues (Sahoo et al., 2016), and ProSA finally confirms the validity of the model by showing a quality score plot, calculated by comparing the input model using RCSB as a reference database (Wiederstein and Sippl, 2007). After satisfying all constraints of assessment, models proceeded for docking analysis. For docking analysis, all target proteins and ligands were prepared as PDB format. Melatonin was first converted to PDBqt format using AutoDock Tools (1.5.6rc2). Both protein and ligand were then passed through AutoDockVina, which is a docking software that interprets docking results in the form of binding energies (*E*-value). The well-docked pose of ligand in each target protein was further analyzed by DSV in term of ligand pose orientation and molecular interactions.

### Statistical Analysis

The 2D gel spots, western blot bands, and morphological data were analyzed using ImageJ software (Image J 1.30)^5^. Data are presented as means ± standard error of mean. Data were analyzed by one-way analysis of variance followed by post-hoc Bonferroni multiple comparison tests using the graph-pad prism-5 software. Symbol ^∗^ or ^#^ represents a significant difference with a *p*-value of < 0.05; symbol ^∗∗^ or ## represents a significant difference with a *p*-value of < 0.01; and symbol ^∗∗∗^ or ### represents a significant difference with a *p*-value of < 0.001. The symbol ^∗^ indicates a significant difference compared with the sham group, and ^#^ indicates a significant difference compared with the MCAO group.

## Results

### Effects of Melatonin on Apoptosis and Neurodegeneration

**FIGURE 1:**
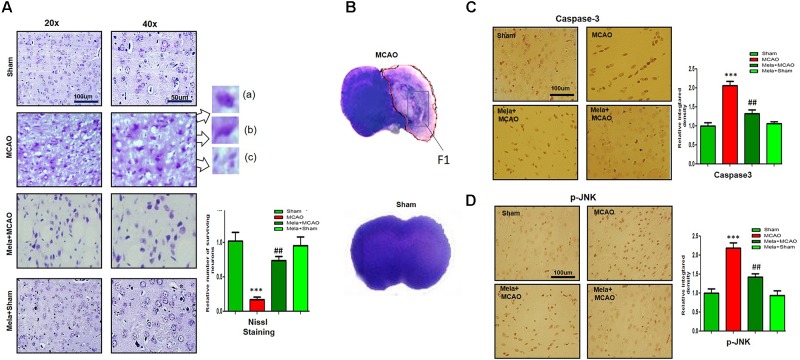
Representative photomicrographs of cresyl violet staining show the extent of surviving neurons in the striatum **(A)**. The number of experiments performed = 3. ^∗∗∗^*p* < 0.001 indicates a significant difference compared with the sham group; ^##^*p* < 0.01 indicates a significant difference compared with the MCAO group. (a) Necrotic neurons with scalloped and shrunken appearance, intense cytoplasmic eosinophilia, and nucleus basophilia. Such changes are characteristics of eosinophilic necrosis (referred to as red neurons, showed by 40×). (b) Cytoplasmic fading of neurons invariably occurs at later stages of neuronal necrosis. The ghost neurons are large with no definite outline, and nuclei are shown. (c) Some of the inflammatory cells can be observed with rounded shaped oligodendrocytes and microglia near necrotic neurons. **(B)** Nissl staining of coronal sections shows hyperchromatic cortex and striatum, separated by red borderline from the corresponding contralateral brain, 24 h after permanent MCAO. The analyzed striatal region is indicated by square F. **(C,D)** Immunoreactivity of Caspase 3 and p-JNK in the striatum (*N* = 5 rats/group). ^##^Represents a significant difference with a *p*-value of < 0.01, and ^∗∗∗^ represents a significant difference with a *p*-value of < 0.001. ^∗^Indicates a significant difference compared with the sham group, and ^#^ indicates a significant difference compared with the MCAO group.

Nissl staining was used to distinguish between necrotic and intact neurons in the striatum and to examine the neuroprotective effect of melatonin. A substantial difference was observed 24 h after permanent ischemia in the striatum between MCAO and sham-operated animals (*p* < 0.001) (Figure 1A). Robust neuronal changes were found in this highly prone area in the brain, and melatonin pretreatment attenuated these changes. Aberrant morphological features, including changes in neuronal size and shape (swelling and scalloped angular nature), alteration in color (cytoplasmic eosinophilia/pyknosis, nuclear basophilia), and vacuolation (swollen and shrunk appearance of neurons), were observed in the striatum in MCAO rats compared with sham-operated rats (Figures 1A,B). Histological analysis did not find noticeable alterations in sham-operated animals. Significantly more intact neuronal cells were found in the melatonin-treated group than in the MCAO-operated group (*p* < 0.01, Figure 1A). Activated JNK is linked to neuronal apoptosis by mediating caspase activation (Yang et al., 2009). JNK activation leads to more apoptosis in the ischemic striatum than in the ischemic cortex due to significant overexpression of JNK in the striatum (Okuno et al., 2004). In this study, activated JNK and caspase-3 were observed in the striatal tissue in the MCAO group compared with the sham-operated group (*p* < 0.001, Figures 1C,D). Notably, treatment with melatonin reversed the activation and significantly reduced the expression levels of p-JNK and caspase-3 (*p* < 0.01).

**FIGURE 2:**
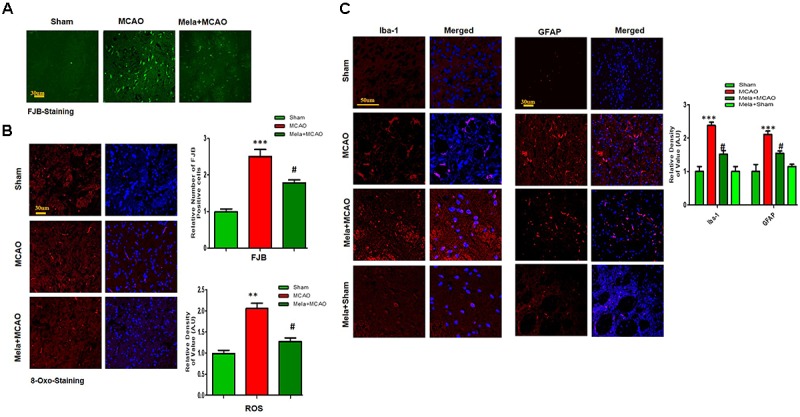
Representative images of FJB **(A)** and 8-oxoguanine **(B)** staining. Each experiment was performed 3 times (*n* = 5 per group). ∗∗∗Shows a significant difference compared with sham rats; ^#^ shows a significant difference compared with MCAO rats. ∗∗∗*p* < 0.001, ^∗∗^*p* < 0.01, and ^#^*p* < 0.05. **(C)** Melatonin attenuated MCAO-triggered activation of astrocytes and microglia. Immunoreactivity of astrocytes (GFAP-positive cells) and microglia (Iba-1-positive cells) in sham, ischemic, and melatonin-operated groups is shown. Scale bar = 30 μm or 50 μm. The GFAP- and Iba-1-positive cells were visualized by TRITC. ^∗∗∗^Represents a significant difference with a *p*-value of < 0.001, and ^#^ represents a significant difference with a *p*-value of < 0.05. ^∗^Shows a significant difference compared with sham rats, and ^#^ shows a significant difference compared with MCAO rats.

Fluoro-Jade B (FJB) is a convenient marker of neuronal degeneration. Melatonin-treated rats (Mela + MCAO) showed relatively intact neuronal morphology, which was comparable to that in sham-operated rats (Figure 2A). In contrast, severe neuronal degeneration (strong FJB staining) was observed in the striatum in MCAO-operated rats (*p* < 0.001; Figure 2A). Studies have consistently demonstrated increased free radical generation during ischemic damage, and the increased free radicals promote the breakage of BBB and facilitate cytotoxic edema. Therefore, we next determined the generation of reactive oxygen species (ROS) in the striatum using fluorescent 8-oxoguanine as an oxidative stress marker. The results showed higher expression of 8-oxoguanine in the ischemic tissues than in sham control tissues (*p* < 0.01). Notably, treatment with melatonin attenuated the ischemia-induced oxidative stress (*p* < 0.05, Figure 2B).

### Melatonin Treatment Attenuates MCAO-Induced Reactive Gliosis

Ischemic stroke is characterized by reactive gliosis, in which astrocytes and resident glial cells are upregulated to mediate the progression of ischemic injury. The activated hypertrophic cells work as a resident machinery to generate inflammatory mediators. Because these cells are primarily involved in neuroinflammation and neurodegeneration, we investigated the neuroprotective effect of melatonin on astrocyte (GFAP-reactive cells) and microglial activation (Iba-1-reactive cells) in the ischemic striatum. Immunofluorescence analysis revealed significant increases in GFAP- and Iba-1-reactive cells in the striatum in the MCAO group compared with the sham group (*p* < 0.001, Figure 2C). Melatonin treatment significantly decreased the number of these hyperactive cells in the striatum (*p* < 0.05).

### Differential Expression of Proteins

**FIGURE 3:**
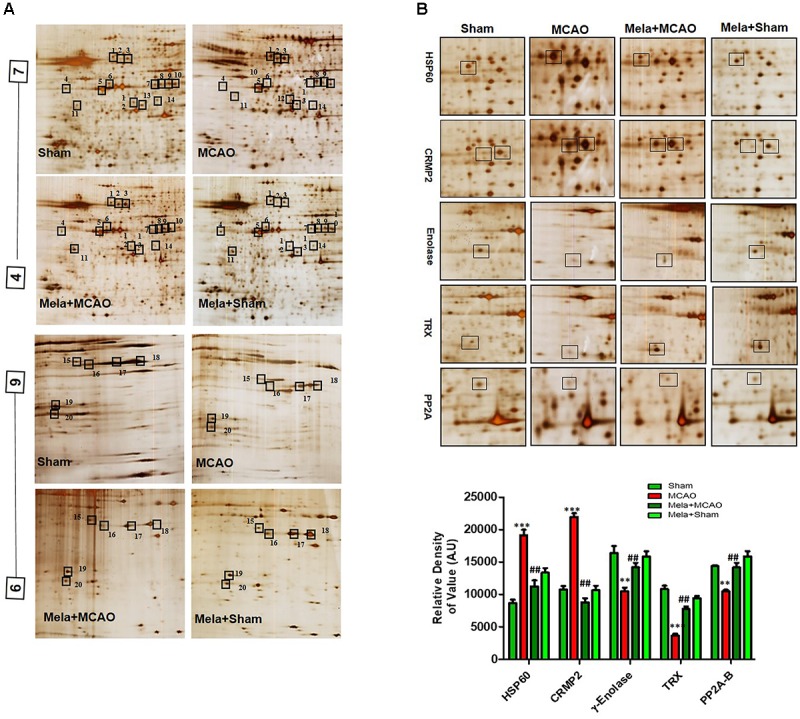
Two-dimensional gel electrophoresis maps. **(A)** Isoelectric focusing was performed at pH 4–7 and 6-9 using IPG strips. The IPG strips were then loaded on a gradient SDS gel and stained with silver nitrate. The spots were identified using MALDI-TOF and NCBI as reference databases. Squares indicate the protein spots that were differentially expressed among different experimental groups. **(B)** Magnified protein spots of HSP60, CRMP2, γ-enolase, TRX, and PP2A. Protein spots were analyzed using ImageJ software. Densitometric analysis is expressed in arbitrary units as means ± SEM for the indicated proteins (*n* = 5 per group). Symbols ^∗∗^ or ## and ^∗∗∗^ or ### represent significant differences with *p* < 0.01 and *p* < 0.001, respectively.

Electrophoretic protein maps were constructed after peptide analysis by mass spectrometry, and protein spots were clearly identified by MALDI-TOF analysis (Figure 3A and Table 1). Five proteins were selected for further analysis (Figure 3B). Proteins were selected based on several factors, including antibody availability, literature accessibility, and relative roles of proteins in ischemic brain injury. It is believed that γ-enolase, CRMP2, HSP60, TRX, and PP2A have vital roles in ischemic stroke because these proteins are largely involved in metabolism, hemostasis, and neuronal sprouting. The upregulated expressions of γ-enolase, CRMP2, HSP60, TRX, and PP2A, identified by MALDI-TOF analysis, were observed in the MCAO group (Figure 3B), but the upregulation of these proteins was significantly attenuated in the melatonin-treated group, indicating the neuroprotective effect of melatonin in the ischemic model.

**Table 1:** List of proteins that were differentials expressed.

Spot	Protein name	Accession	M.W	PI	Mass	Sequence
no.		no.			matched	coverage (%)
1	60 kDa heat shock protein	P63039	60917	5.91	11/133	32%
2	Dihydropyrimidinase-related protein 2	P47942	83856	6.64	7/109	29%
3	Dihydropyrimidinase-related protein 2	P47942	62278	6	24/103	52%
4	γ-enolase	P07323	47141	5	14/70	34%
5	Prolactin-8A5 isoform XI	P33579	27267	5.47	7/97	22%
6	Eukaryotic initiation factor 4A-II	Q5RKI1	46373	5.33	16/82	40%
7	Succinyl-CoA ligase subunit beta	Q9Z219	50274	7.75	11/74	24%
8	Adenosine kinase	Q64640	40108	5.72	12/95	41%
9	MAP kinase kinase	Q01986	43465	6.18	12/72	30%
10	Adenosylhomocysteinase	P10760	47507	6.07	15/132	33%
11	Thioredoxin	Q920J4	3223	4.84	8./87	42%
12	Isocitrate dehydrogenase[NAD+] subunit alpha	Q99NA5	39588	6.47	8/93	31%
13	NAD-specific Isocitrate dehydrogenase[NAD+] subunit alpha	Q99NA5	39588	6.47	8/93	31%
14	Protein phosphatase 2A, regulatory subunit B	P36877	36594	5.88	9/56	29%
15	Alchol dehrdogenase	P51635	36510	6.8	9/104	28%
16	Glyceraldehyde-3- phosphate dehydrogenase	P04797	35828	8.14	16/106	46%
17	Glyceraldehyde-3- phosphate dehydrogenase	P04797	35828	8.14	20/94	62%
18	Glyceraldehyde-3- phosphate dehydrogenase	P04797	35828	8.14	16/75	55%
19	Nucleoside diphosphate kinase B	P19804	17283	6.9	8/86	49%
20	Peroxiredoxin-5	Q9R063	22165	8.94	9/114	46%

### Validation of Proteins Downregulated After MCAO Injury

**FIGURE 4:**
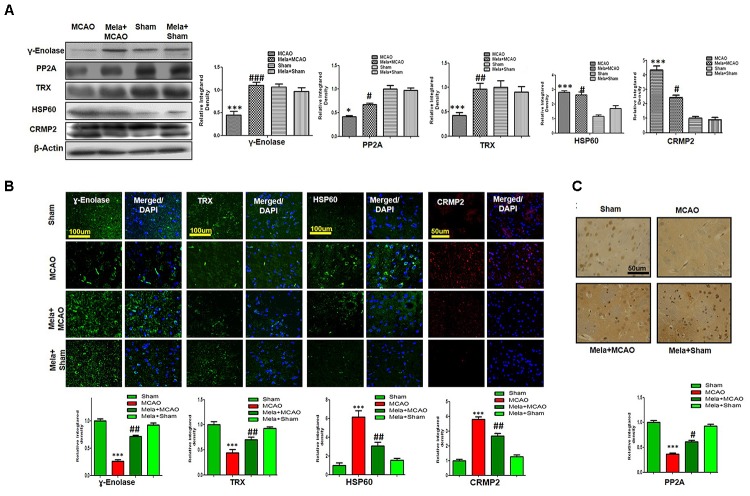
Western blot analysis of **(A)** γ-enolase, HSP60, CRMP2, TRX, and PP2A in the striatum in the sham-operated, MCAO, and melatonin-treated MCAO groups. The bands were quantified using ImageJ and analyzed by graph-pad prism-5 software. β-actin was used as a control. Densitometric analysis is expressed in arbitrary units as means ± SEM for the indicated proteins. Symbol ^∗^ or ^#^ represents a significant difference with *p* < 0.05; symbol ^∗∗^ or ^##^ represents a significant difference with *p* < 0.01; symbol ^∗∗∗^ or ^###^ indicates a significant difference with *p* < 0.001. The symbol ^∗^ indicates a significant difference compared with the sham group, and ^#^ indicates a significant difference compared with the MCAO group. (B) Immunofluorescence reactivity of γ-enolase, TRX, HSP60, and CRMP2 was examined in the striatum (*N* = 5 rats/group). The data are representative of 3 independent experiments. Magnification, 40×. Scale bar = 100 or 50 μm. These proteins showed cytoplasmic localization. HSP60, TRX, and γ-enolase were visualized by FITC, and CRMP2 was visualized by Alexa-Flour 594. **(C)** Immunohistochemical staining of PP2A in the striatum. Symbols ^##^ and ^#^ represent significant differences with *p* < 0.01 and *p* < 0.05, respectively; Symbol ^∗∗∗^ or ^###^ represents a significant difference with *p* < 0.001. The symbol ^∗^ indicates a significant difference compared with the sham control group, and ^#^ indicates a significant difference compared with the MCAO group.

Enolases have pivotal roles in energy metabolism, signifying the importance of enolases in stroke (Sarnat, 2013). Proteomic analysis revealed abundance of γ-enolase (*p* < 0.01), TRX (*p* < 0.001), and PP2A after MCAO injury (*p* < 0.01, Figure 3B). Moreover, we examined the expression levels of these proteins using western blot analysis with β-actin as a loading control, and significant differences in expression levels of these proteins were observed between MCAO and other experimental groups (Figure 4A). Furthermore, we performed immunofluorescence to investigate the distribution of these proteins in the ischemic striatum (Figure 4B). The findings further indicated that the expression levels of γ-enolase and TRX decreased in the striatum in the ischemic brain in the MCAO group (*p* < 0.001, Figure 4B), but melatonin treatment attenuated the decrease of these proteins in the Mela + MCAO group (Figure 4B, *p* < 0.01). In addition, immunohistochemical staining validated the proteomic findings. Notably, immunohistochemical staining revealed that the number of PP2A subunit B-positive cells decreased in the ischemic striatum (Figure 4C, *p* < 0.001), and melatonin treatment significantly recovered the PP2A expression levels in the Mela + MCAO group (Figure 4C, *p* < 0.05).

### Validation of Proteins Upregulated After MCAO Injury

In this study, we also identified proteins with increased expression levels after MCAO injury. The proteomic analysis revealed an increased abundance of CRMP2 and HSP60 (*p* < 0.001) after MCAO (Figure 3B). In addition, western blotting analysis was performed with an equivalent quantity of protein samples from different experimental groups using β-actin as a loading control (Figure 4A), and the results confirmed significant differences in band intensities of CRMP2 and HSP60 between MCAO and sham groups (*p* < 0.001, Figure 4A). Moreover, immunofluorescence analysis further validated the increased expression of these proteins after MCAO (*p* < 0.001, Figure 4B). CRMP2 displayed proteolytic cleavage after MCAO, and western blotting analysis of CRMP2 showed a migration pattern of cleaved bands (Figure 4A). The intact mass of CRMP2 is 66 kD and can degrade to 62 and 55 kD proteins (Zhang et al., 2007). Western blotting analysis showed an elevated expression of cleaved 55 kD CRMP2 in the MCAO group.

### Homology Modeling and Validation Process

**FIGURE 5:**
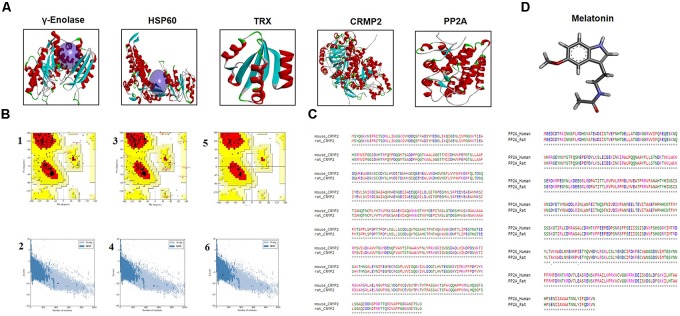
**(A)** The representative tertiary structures of proteins (γ-enolase, HSP60, CRMP2, TRX, and PP2A). **(B)** Homology modeling and validation of tertiary structures. Ramachandran plots for γ-enolase (1), HSP60 (3), and TRX (5) are shown. ProSA findings of γ-enolase, HSP60, and TRX are represented by (2), (4), and (6), respectively. **(C)** Representative sequence alignment of CRMP2 (rat accession number P47942) with mouse CRMP2 (PDB code:5UQC) and PP2A (UniProt accession number P36877) with human PP2A (PDB ID: 3DW8) by clustal omega. **(D)** The ligand melatonin structure was drawn in ChemSketch and was converted to PDB format by Pymol.

The BLASTp analysis of γ–enolase, HSP60, and TRX sequences identified chain A of human γ-enolase 2 in complex with phosphonoacetohydroxamate (PDB ID: 4ZA0), chain A of mitochondrial chaperonin symmetrical ‘football’ complex (PDB ID: 4PJ1), and chain A crystal structure of catalytic domain of a new human thioredoxin-like protein (PDB: 1GH2) as the best templates with sequence identity of 98%, respectively (Figure 5A). A total of 5 models were generated for each target protein by the automated SWISS-MODEL server (Biasini et al., 2014). The chosen models were subjected to energy minimization via molecular dynamics simulation in GROMACS v5.0.6. The energy of each protein was minimized with CHARMm27 force-field parameterization and by applying the steepest descent algorithm at force 10.0 kJ/mol for 50000 steps. The potential energies of minimized γ-enolase, HSP60, and TRX were calculated as -8.0 × 106 kJ/mol, -2.2 × 106 kJ/mol, and -2.2 × 106 kJ/mol, respectively. The final 3D structure is shown in Figure 5A. The stereochemical integrity of the energy-minimized models was evaluated using Procheck. Ramachandran plot distributes the amino acid residues of γ-enolase, HSP60, and TRX models into respective regions as shown in Figures 5B1,B3,B5. Briefly, the amino acid residues were 90.7% and 91.1% in the favored region, 8.8 and 6.9% in the allowed region, and 0.3 and 1.9% in the generously allowed region for γ-enolase and HSP60 models, respectively. In the TRX analysis, the amino acid residues were 95.8 and 4.2% in the favored and allowed regions, respectively. These results suggest that the backbone dihedral angles of both models are reasonably accurate. Moreover, the models were further validated by ProSA server for potential errors (Wiederstein and Sippl, 2007). ProSA is used to determine the refinement of protein structures in term of *Z*-score and residue energies. The *Z*-score generally shows the quality of the model, and negative values of residue energies confirm the uniformity of the model. *Z*-scores of γ-enolase (-9.95), HSP60 (-10.95), and TRX (-6.5) are depicted in Figures 5B2,B4,B6. The findings suggest that the generated 3D models are of good quality (Wiederstein and Sippl, 2007).

Notably, the BLASTp of CRMP2 (UniProt accession number P47942) and PP2A (UniProt accession number P36877) identified mouse CRMP2 structure (PDB code: 5UQC) and human PP2A (PDB ID: 3DW8) as the best identical sequence templates (100% sequence identity) in RCSB, respectively (Figure 5A). Furthermore, we aligned these protein sequences by Clustal Omega (Chenna et al., 2003), and the findings revealed that entire sequences of these proteins were identical and conserved (Figure 5C). Therefore, we used mouse-CRMP2 and human PP2A as structure analogs of rat-CRMP2 and rat PP2A for docking studies, respectively. Moreover, the structure of melatonin was retrieved from Pubchem database^6^, and its 2D structure, which was drawn in ChemSketch, was converted to 3D structure and saved as PDB file in DSV (Figure 5D).

### Docking Studies

**FIGURE 6:**
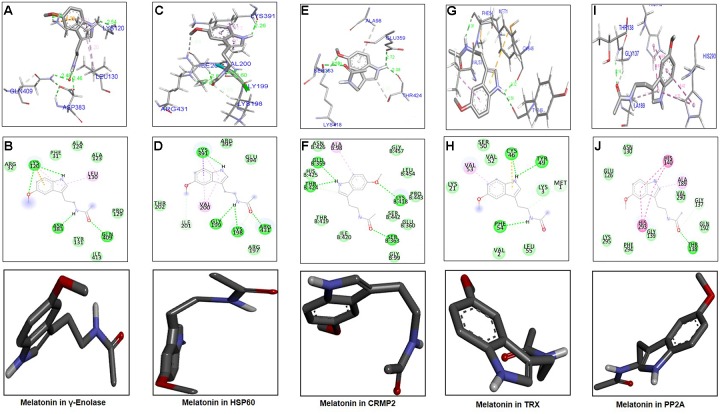
Docking results and best pose of melatonin that fits γ-enolase, HSP60, CRMP2, TRX, PP2A. Post-docking analysis were visualized by DSV in both 2D and 3D poses. Interaction between melatonin and γ-enolase were shown by **A,B**, HSP60 by **C,D**, CRMP2 **(E,F)**, TRX **(G,H)**, and PP2A by **I,J**. 3D poses were shown by **A,C,E,G,I** and 2D by **B,D,F,H,J**.

The ligand melatonin was docked in the catalytic active pocket of HSP60, γ-enolase, CRMP2, TRX, and PP2A. Table 2 shows the binding energies, and Figure 6 represents the best pose of melatonin that fits γ-enolase, HSP60, CRMP2, TRX, and PP2A after docking analysis. The docking analysis showed that melatonin fitted with γ-enolase with a bond distance of 2.46–2.54Å, indicating high polar contacts (Figures 6A,B). Furthermore, it was observed that four hydrogen bonds were formed between melatonin and γ-enolase. Two hydrogen bonds were formed between Lys120 and melatonin. Nitrogen in pyrrole ring is hydrogen bond donor, while oxygen of methoxy group acts as hydrogen bond acceptor. Similarly, two hydrogen bonds were formed between the amide group (consisting of both hydrogen bond donor and acceptor) and amino acids including Asp383 and Gln409 (Figure 6B). In addition, Van der Waal and electrostatic interactions, which further provide stability to melatonin binding in the γ-enolase active site (Bosshard et al., 2004), were also observed.

**Table 2:** Binding energy values or binding affinity (*E*-value), expressed as Kcal/mol of Gamma enolase (γ-Enolase), heat shock protien-60 (HSP-60), collapsin response mediated protein 2 (CRMP2), Thioredoxin (TRX), and Protein phosphatase 2A (PP2A).

Protein	Energy values after
	docking (kcal/mol)
γ-Enolase	-5.5
HSP60	-5.9
CRMP2	-6.2
TRX	-5.4
PP2A	-6.6


The docking results of melatonin and HSP60 are shown in Figures 6C,D. Four hydrogen bonds were observed between melatonin and HSP60, and three were formed by the amide group. Other residues including Arg395, Glu394, Arg197, and Thr202 had Van der Waal interactions with melatonin (Figures 6C,D). Post-docking interactions for CRMP2 are shown in Figures 6E,F. Melatonin fits with CRMP2 with a bond distance of 2.22–2.72Å. All kinds of polar and other electrostatic interactions were visualized in DSV. In addition, the docking results of TRX (Figures 6G,H) and PP2A (Figures 6I,J) are also shown in this study. All these analyses examined the most likely binding patterns of melatonin with each target protein.

## Discussion

In this study, we performed a comparative analysis of differentially expressed proteins in the ischemic striatum between MCAO and Mela + MCAO rats. The focus of this study was to determine the protein profile of the rat striatal tissue after MCAO injury, to perform bioinformatics analysis, and to examine the effect of melatonin. The quantitative 2D-MS technique was used to illustrate the differential expression proteins in the striatal tissues in the MCAO adult rats.

Because no 3D structures for γ-enolase, HSP60, and TRX have been reported for rats in the PDB data bank, the homology modeling was performed to build 3D structures of these proteins (Burley, 2000). The stability of these structures was further assessed by MD simulation. MD simulation is an *in silico* modeling method for studying movements of particles (mostly atoms). MD simulation is used to infer the real behavior of atoms under the specified environmental conditions and is a well-practiced discipline in biological systems, where MD can be used to investigate the stability and physiological orientation and/or confirmation of bio-molecules. In this study, we used GROMACS v5.0.6 package to minimize the energy of each modeled structure. We found that rat CRMP2 and PP2A showed 100% sequence coverage with mouse CRMP2 (PDB^#^ 5UQC) and human PP2A (PDB ID: 3DW8) respectively, and the sequences were further aligned by clustal omega (Figure 2B). Omega analysis suggests that sequences of CRMP2 and PP2A in these species are conserved. Melatonin structure was drawn in ChemSketch and converted to PDB format by Pymol. Docking analysis was performed by AutodockVina, and binding energy was evaluated. The interaction of docked pose of ligand (melatonin) in protein was further visualized by discovery studio (DS). Weak intermolecular interactions play an important role in stabilizing the ligand energetically in the target protein. Therefore, we identified the molecular interaction patterns of melatonin binding in each target protein. Molecular interactions were elaborated in terms of hydrogen bonding, Van der Waals forces, and electrostatic interactions. Our thorough analysis of computational docking indicates that melatonin binds each target protein by H-bonds and other hydrophobic interactions. Therefore, we speculate that the formation of H-bonds between the corresponding protein and melatonin supports the correspondent complex stability.

The molecular mechanism of ischemic brain damage is characterized by complicated pathophysiology. Brain ischemia leads to a cascade of events, such as glutamate excitotoxicity, energy failure, and formation of toxic radicals. The proteomic study helps identify proteins that are imperative in brain injury and provides an in-depth analysis of the mechanism underlying neuronal degeneration following ischemic stroke. In this study, we identified 5 proteins that were differentially expressed in the ischemic brain between MCAO and mela + MCAO groups.

CRMPs are a group of cytoplasmic proteins (CRMP1, CRMP2, and CRMP5) and have important roles in neuronal polarization and axonal growth (Quach et al., 2015; Zhang and Koch, 2017). Expression of CRMP2 is largely depending upon the underlying neurological diseases (Czech et al., 2004; Li et al., 2018). The biological role of CRMP2 in ischemic brain injury has remained unclear. Some studies suggest increased expression of CRMP2 in the ischemic brain, but other studies indicate reduced expression of this protein (Chen et al., 2007; Shah et al., 2014; Yang et al., 2016). Moreover, consistent findings suggest that intact CRMP2 (66 kDa) is degraded into 55 kDa break down product (BDP) proteins by calpain-mediated proteolysis during brain injury (Yoshimura et al., 2005). These studies also identify different CRMP2 isoforms with varied pI values (Franzen et al., 2003). However, we identified two CRMP2 spots with the same pI values and molecular weight. The discrepancy could be attributed to species and method variability. Several studies have shown the entanglement of CRMP2 in Ca^2+^ signaling (Chi et al., 2009; Brittain et al., 2011). Sequestration of CRMP2-Ca^2+^ signaling attenuates inflammation in both stroke and neuropathic pain model (Brittain et al., 2011, 2012). Moreover, altered expression of CRMP2 has been previously demonstrated in Alzheimer’s disease, Parkinson’s disease, alcohol-induced neurodegeneration, and traumatic brain injury (Barzilai et al., 2000; Kobeissy et al., 2006; Matsuda-Matsumoto et al., 2007). CRMP2 is also implicated in neurogenesis and plasticity because it promotes axonal regeneration and inhibits the generation of p53-induced apoptotic genes (Suzuki et al., 2003; Llanos et al., 2006). CRMP2 is a downstream target of GSK3β, which can antagonize the polarization activity of CRMP2 by phosphorylation (Yoshimura et al., 2005). In this present study, we found two different spots for CRMP2, which were differentially regulated in the ischemic striatum and sham-operated control striatum. Moreover, melatonin treatment maintained the expression level of intact CRMP2 in the Mela + MCAO group. Western blotting analysis indicates that increased production of 55 kDa breakdown product (BDP) is associated with ischemic injury, and melatonin treatment attenuates the increase of the BDP and maintains the integrity of intact CRMP2.

Heat shock proteins are physiological sensors and are localized largely in mitochondria with a little fraction in the cytosol. Several studies suggest that overexpression of HSP60 induces mitochondrial biogenesis during ischemic damage, indicating the intrinsic protective mechanism of the brain during stressful settings (Bertoni-Freddari et al., 2006; Gutsaeva et al., 2006; Yin et al., 2008; Truettner et al., 2009). HSP60 dimerizes with HSP10, playing an integral role in protein folding and import and thus helping maintain the structural and functional integrity of mitochondria. Furthermore, the inflammatory role of HSP60 has been also demonstrated in various brain injuries, where HSP60 binds to toll-like receptors (TLRs) in microglia cells in the brain and protects against disturbances in the neuronal environment (Lehnardt et al., 2008). Because of the significant role in neuroinflammation, HSP60 has been considered a promising biomarker of neuronal injury (Chang et al., 2012). HSP60 has both survival and apoptotic functions largely depending on its localization (Kim et al., 2009). Coupling of HSP60 to TLR4 and Bax leads to apoptotic (NF_K_B activation) and survival pathways in the cardiac tissue, respectively (Lin et al., 2007; Wang et al., 2010). Our proteomic analysis suggests overexpressed HSP60 in the ischemic brain, and the findings are consistent with previous observations (Hwang et al., 2007; Yin et al., 2008). Furthermore, melatonin treatment maintains the expression level of HSP60. In agreement to the previous findings, the induction of HSP60 in the present study indicates the intrinsic protective activity of the brain after ischemic brain damage.

γ-enolases/enolase-2 is abundantly present in mature neurons in the white and gray matters, and one recent study has regarded γ-enolases as a biochemical marker of brain injury (Sahu et al., 2017). Previous studies have demonstrated that γ-enolase is a neurotropic agent, and it can enhance neuronal survival and promote axonal growth (Hafner et al., 2012). γ-enolases are very important for energy generation during glycolysis, and deterioration in γ-enolase activity would adversely affect energy metabolism in the brain. The glycolytic functions of γ-enolases and other metabolic enzymes are significantly impaired by ischemic damage. Notably, downregulation of γ-enolase leads to neurodegeneration (Kilic et al., 2005). However, a high concentration of γ-enolase is found in various brain pathologies, and inhibition of γ-enolase attenuates inflammation-related cellular injury (Haque et al., 2017). Our study showed that NSE expression decreased 24 h after cerebral ischemia, and the results are similar to previous findings (Gim et al., 2015; Jeon et al., 2017; Park et al., 2018). Our findings are further verified by western blot analysis and confocal immunofluorescence analysis, and melatonin treatment prevents the ischemia-induced decrease of NSE. γ-enolases exert pleiotropic action and mediate neuronal repair, axonal outgrowth, and neurotrophic activity by PI3K and MAPK pathways (Hafner et al., 2012; Polcyn et al., 2017). Our findings suggest that preservation of γ-enolase during MCAO contributes to the neuroprotective effect of melatonin, but the relation between γ-enolase and melatonin is not yet well established. Therefore, future work is needed to comprehensively investigate the underlying neuroprotective mechanism.

Thioredoxin (TRX) has multi-biological functions including redox signaling. TRX participates in the eradication of ROS such as hydrogen peroxide and other toxic radicals (Landino et al., 2004; Messens and Silver, 2006). TRX is an important component of the TRX system, in which TRX and peroxiredoxin act as anti-oxidant enzymes and maintain a hemostatic-reduced environment. Downregulation of thioredoxin leads to apoptotic death by activating ASK1, and apoptosis further stimulates stress signaling kinases such as JNK. In addition, upregulation of thioredoxin impedes ASK1-induced apoptosis (Lee et al., 2008). A number of studies have consistently demonstrated that ischemic brain damage is linked to free radical generation, which triggers neuronal damage by apoptosis and necrosis. Moreover, free radical scavengers protect neuronal cells from ischemic damage by attenuating free radical formation. In fact, the exogenous administration of these scavengers attenuates neuronal degeneration during ischemic stroke (Wang et al., 2015). During ischemic brain injury, ROS are generated via the hyperactivated electron transport chain and the NADPH oxidase system and are implicated in the detrimental effects on DNA, cell membrane, ion channels, and redox signaling (Valko et al., 2007). Protective endogenous enzymes, such as TRX, play a significant role in the prevention of the oxidative stress-induced neuronal damage (Chan, 2001). TRX isoforms are localized in both cytosol and mitochondria with varying degrees of antioxidant capacity. Several studies have demonstrated enhanced tolerability of ischemic brain with overexpressed TRX (Stroev et al., 2004). Moreover, TRX is demarcated as an oxidation, inflammation, and immune deregulatory marker due to the substantial role (Al-Gayyar et al., 2011; Zhang et al., 2012). Furthermore, oxidative stress and ROS are hallmarks of several neurodegenerative disorders, further strengthening the anti-oxidant capacity of TRX because the brain has a poor catalase and glutathione activity (Erecińska and Silver, 2001). Several studies have demonstrated that TRX release is controlled by NRF2 transcriptional factors, which activate antioxidant genes, including TRX, after translocation to the nucleus (Wu et al., 2015). Moreover, one recent study by Ali et al. (2018) indicates that melatonin activates NRF2 in the brain. Our proteomics study revealed decreased expression of TRX in the striatal tissue in MCAO-operated rats, and melatonin treatment attenuated this decline of TRX triggered by ischemic injury, indicating the antioxidant nature of melatonin. However, further studies are needed to explore the exact mechanism.

Protein phosphatase 2A (PP2A) is a serine-threonine phosphatase and has multiple biological activities from cellular metabolism to development and apoptosis. Subunit B of PP2A is ubiquitously expressed in the brain, and it regulates both axonal growth and neurogenesis. Our previous study has found downregulated PP2A in the cortex 24 h after MCAO (Shah et al., 2015). Phosphorylation and de-phosphorylation should be rigidly regulated because this process determines the fate of the signaling cascade in various disease pathologies including neurodegeneration (Sontag et al., 2004), and PP2A is an important component in the de-phosphorylation process (Gong et al., 2000; Liu et al., 2005; Gong and Iqbal, 2008). PP2A maintains tau in dephosphorylated form, which helps in axonal microtubule assembling. Moreover, PP2A downregulation leads to tau hyperphosphorylation, a characteristic hallmark in the Alzheimer’s brain. In addition, PP2A inhibits the induction of stress kinases, such as JNK and p38 (Chen et al., 2009). PP2A also de-phosphorylates NMDA receptor subunits and hinders calcium currents (Ma and Sucher, 2004). Amassing of calcium inside neuronal cells leads to excitotoxicity-induced neuronal death in cerebral ischemia. Our study demonstrated decreased expression of PP2A subunit B in the ischemic striatum. Moreover, the attenuated expression of PP2A is implicated in ischemic brain injury, as demonstrated by Koh (2012). In addition, immunohistochemical staining further validates the proteomics findings that PP2A subunit B-positive cells decrease in the ischemic striatum. Moreover, treatment with melatonin restores PP2A expression. Our results are consistent to previous observations that melatonin mitigates ischemic injury-induced downregulation of PP2A in the ischemic and AD animal models, as well as in *in vitro* models (Koh, 2013; Arribas et al., 2018).

In summary, melatonin attenuates MCAO-induced neuronal damage by modulating the expression of proteins involved in energy metabolism, homeostasis, axonal growth, and oxidative response in the striatum. The study suggests that melatonin may potentially ameliorate the ischemic stroke damage by modulating expression of several proteins in the striatum. Moreover, a more in-depth understanding of the functions of proteins (CRMP2, HSP60, Enolase, TRX, and PP2A) could provide new opportunities for treating a wide range of neurodegenerative disorders, including ischemic stroke.

## Data Availability Statement

The authors here by state that the datasets generated in this study will be available upon request from the corresponding author.

## Author Contributions

FS designed, managed the experimental work, and wrote the manuscript. FS, TA, MF, TM, SA, and KS performed the Western blot and morphological experiments, FS, TA, P-OK, and MK arranged the data and performed the data analysis. AZ and KL performed the bioinformatics analysis. MK is a corresponding author, reviewed and approved the manuscript and holds all the responsibilities related to this manuscript. All authors reviewed the manuscript.

## Conflict of Interest Statement

The authors declare that the research was conducted in the absence of any commercial or financial relationships that could be construed as a potential conflict of interest.

## Footnotes

1 http://www.uniprot.org

2 https://swissmodel.expasy.org/

3 https://www.ebi.ac.uk/thornton-srv/software/PROCHECK/

4 https://prosa.services.came.sbg.ac.at/prosa.php

5 https://imagej.nih.gov/ij/

6 https://pubchem.ncbi.nlm.nih.gov/search/

## References

[B1] Abraham M. J. Murtola T. Schulz R. Pall S. Smith J. C. Hess B. (2015). GROMACS: high performance molecular simulations through multi-level parallelism from laptops to supercomputers. *SoftwareX* 1 19–25. 10.1016/j.softx.2015.06.001

[B2] Al-Gayyar M. M. Abdelsaid M. A. Matragoon S. Pillai B. A. El-Remessy A. B. (2011). Thioredoxin interacting protein is a novel mediator of retinal inflammation and neurotoxicity. *Br. J. Pharmacol.* 1 170–180. 10.1111/j.1476-5381.2011.01336.x 21434880 PMC3171869

[B3] Ali T. Rehman S. U. Shah F. A. Kim M. O. (2018). Acute dose of melatonin via Nrf2 dependently prevents acute ethanol-induced neurotoxicity in the developing rodent brain. *J. Neuroinflammation* 15:119. 10.1186/s12974-018-1157-x 29679979 PMC5911370

[B4] Alluri H. Wilson R. L. Anasooya Shaji C. Wiggins-Dohlvik K. Patel S. Liu Y. (2016). Melatonin preserves blood-brain barrier integrity and permeability via matrix metalloproteinase-9 inhibition. *PLoS One* 11:e0154427. 10.1371/journal.pone.0154427 27152411 PMC4859489

[B5] Andrabi S. S. Parvez S. Tabassum H. (2015). Melatonin and ischemic stroke: mechanistic roles and action. *Adv. Pharmacol. Sci.* 2015:384750. 10.1155/2015/384750 26435711 PMC4575994

[B6] Arribas R. L. Romero A. Egea J. de Los Ríos C. (2018). Modulation of serine/threonine phosphatases by melatonin: therapeutic approaches in neurodegenerative diseases. *Br. J. Pharmacol.* 10.1111/bph.14365 [Eub ahead of rint]. 29781146 PMC6057903

[B7] Barzilai A. Zilkha-Falb R. Daily D. Stern N. Offen D. Ziv I. (2000). The molecular mechanism of dopamine-induced apoptosis: identification and characterization of genes that mediate dopamine toxicity. *J. Neural. Transm. Suppl.* 60 59–76. 10.1007/978-3-7091-6301-6_4 11205158

[B8] Bertoni-Freddari C. A. Fattoretti P. Casoli T. Di Stefano G. Solazzi M. Perna E. (2006). Reactive structural dynamics of synaptic mitochondria in ischemic delayed neuronal death. *Ann. N. Y. Acad. Sci.* 1090 26–34. 10.1196/annals.1378.003 17384244

[B9] Biasini M. Bienert S. Waterhouse A. Arnold K. Studer G. Schmidt T. (2014). SWISS-MODEL: modelling protein tertiary and quaternary structure using evolutionary information. *Nucleic Acids Res.* 42 252–258. 10.1093/nar/gku340 24782522 PMC4086089

[B10] Bosshard H. R. Marti D. N. Jelesarov I. (2004). Protein stabilization by salt bridges: concepts, experimental approaches and clarification of some misunderstandings. *J. Mol. Recognit.* 17 1–16. 10.1002/jmr.657 14872533

[B11] Brittain J. M. Duarte D. B. Wilson S. M. Zhu W. Ballard C. Johnson P. L. (2011). Suppression of inflammatory and neuropathic pain by uncoupling CRMP-2 from the presynaptic Ca(2) channel complex. *Nat. Med.* 17 822–829. 10.1038/nm.2345 21642979 PMC3219927

[B12] Brittain J. M. Pan R. You H. Brustovetsky T. Brustovetsky N. Zamponi G. W. (2012). Disruption of NMDAR-CRMP-2 signaling protects against focal cerebral ischemic damage in the rat middle cerebral artery occlusion model. *Channels* 6 52–59. 10.4161/chan.18919 22373559 PMC3367672

[B13] Burley S. K. (2000). An overview of structural genomics. *Nat. Struct. Biol.* 7 932–934. 10.1038/80697 11103991

[B14] Chan P. H. (2001). Reactive oxygen radicals in signaling and damage in the ischemic brain. *J. Cereb. Blood Flow Metab.* 21 2–14. 10.1097/00004647-200101000-00002 11149664

[B15] Chang C. C. Lui C. C. Lee C. C. Chen S. D. Chang W. N. Lu C. H. (2012). Clinical significance of serological biomarkers and neuropsychological performances in patients with temporal lobe epilepsy. *BMC Neurol.* 12:15. 10.1186/1471-2377-12-15 22417223 PMC3342103

[B16] Chen A. Liao W. P. Lu Q. Wong W. S. F. Wong P. H. (2007). Upregulation of dihydropyrimidinase-related protein 2, spectrin α II chain, heat shock cognate protein 70 pseudogene 1 and tropomodulin 2 after focal cerebral ischemia in rats—a proteomics approach. *Neurochem. Int.* 50 1078–1086. 10.1016/j.neuint.2006.11.008 17196711

[B17] Chen L. Liu L. Yin J. Luo Y. Huang S. (2009). Hydrogen peroxide-induced neuronal apoptosis is associated with inhibition of protein phosphatase 2A and 5, leading to activation of MAPK pathway. *Int. J. Biochem. Cell Biol.* 41 1284–1295. 10.1016/j.biocel.2008.10.029 19038359

[B18] Chenna R. Sugawara H. Koike T. Lopez R. Gibson T. J. Higgins D. G. (2003). Multiple sequence alignment with the Clustal series of programs. *Nucleic Acids Res.* 31 3497–3500. 10.1093/nar/gkg500 12824352 PMC168907

[B19] Chi X. X. Schmutzler B. S. Brittain J. M. Wang Y. Hingtgen C. M. Nicol G. D. (2009). Regulation of N-type voltage-gated calcium channels (Cav2.2) and transmitter release by collapsin response mediator protein-2 (CRMP-2) in sensory neurons. *J. Cell Sci.* 122 4351–4362. 10.1242/jcs.053280 19903690 PMC2779133

[B20] Czech T. Yang J. W. Csaszar E. Kappler J. Baumgartner C. Lubec G. (2004). Reduction of hippocampal collapsin response mediated protein-2 in patients with mesial temporal lobe epilepsy. *Neurochem. Res.* 29 2189–2196. 10.1007/s11064-004-7025-3 15672539

[B21] Erecińska M. Silver I. A. (2001). Tissue oxygen tension and brain sensitivity to hypoxia. *Respir. Physiol.* 128 263–276. 10.1016/S0034-5687(01)00306-1 11718758

[B22] Fluri F. Schuhmann M. K. Kleinschnitz C. (2015). Animal models of ischemic stroke and their application in clinical research. *Drug Des. Devel. Ther.* 9:3445. 10.2147/DDDT.S56071 26170628 PMC4494187

[B23] Franzen B. Yang Y. Sunnemark D. Wickman M. Ottervald J. Oppermann M. (2003). Dihydropyrimidinase related protein-2 as a biomarker for temperature and time dependent post mortem changes in the mouse brain proteome. *Proteomics* 3 1920–1929. 10.1002/pmic.200300535 14625854

[B24] García J. J. López-Pingarrón L. Almeida-Souza P. Escudero P. García-Gil F. A. Tan D. X. (2014). Protective effects of melatonin in reducing oxidative stress and in preserving the fluidity of biological membranes: a review. *J. Pineal Res.* 56 225–237. 10.1111/jpi.12128 24571249

[B25] Gim S. A. Lee S. R. Shah F. A. Koh P. O. (2015). Curcumin attenuates the middle cerebral artery occlusion-induced reduction in γ-enolase expression in an animal model. *Lab. Anim. Res.* 31 198–203. 10.5625/lar.2015.31.4.198 26755923 PMC4707148

[B26] Gong C. X. Iqbal K. (2008). Hyperphosphorylation of microtubule-associated protein tau: a promising therapeutic target for Alzheimer disease. *Curr. Med. Chem.* 15 2321–2328. 10.2174/092986708785909111 18855662 PMC2656563

[B27] Gong C. X. Lidsky T. Wegiel J. Zuck L. Grundke-Iqbal I. Iqbal K. (2000). Phosphorylation of microtubule-associated protein tau is regulated by protein phosphatase 2A in mammalian brain implications for neurofibrillary degeneration in Alzheimer’s disease. *J. Biol. Chem.* 275 5535–5544. 10.1074/jbc.275.8.5535 10681533

[B28] Gutsaeva D. R. Suliman H. B. Carraway M. S. Demchenko I. T. Piantadosi C. A. (2006). Oxygen-induced mitochondrial biogenesis in the rat hippocampus. *Neuroscience* 137 493–504. 10.1016/j.neuroscience.2005.07.061 16298077

[B29] Hafner A. Obermajer N. Kos J. (2012). γ-Enolase C-terminal peptide promotes cell survival and neurite outgrowth by activation of the PI3K/Akt and MAPK/ERK signalling pathways. *Biochem. J.* 443 439–450. 10.1042/BJ20111351 22257123

[B30] Haque A. Capone M. Matzelle D. Cox A. Banik N. L. (2017). Targeting enolase in reducing secondary damage in acute spinal cord injury in rats. *Neurochem. Res.* 42 2777–2787. 10.1007/s11064-017-2291-z 28508172 PMC5685929

[B31] Hwang I. K. Ahn H. C. Yoo K. kimkim Y. Lee J. Y. Suh H. W. (2007). Changes in immunoreactivity of HSP60 and its neuroprotective effects in the gerbil hippocampal CA1 region induced by transient ischemia. *Exp. Neurol.* 208 247–256. 10.1016/j.expneurol.2007.08.017 17931626

[B32] Jeon S. J. Kim M. O. Shah F. A. Koh P. O. (2017). Quercetin attenuates the injury-induced reduction of γ-enolase expression in a middle cerebralartery occlusion animal model. *Lab. Anim. Res.* 33 308–314. 10.5625/lar.2017.33.4.308 29399028 PMC5792532

[B33] Kilic U. Kilic E. Reiter R. J. Bassetti C. L. Hermann D. M. (2005). Signal transduction pathways involved in melatonin-induced neuroprotection after focal cerebral ischemia in mice. *J. Pineal Res.* 38 67–71. 10.1111/j.1600-079X.2004.00178.x 15617539

[B34] Kim S. C. Stice J. P. Chen L. Jung J. S. Gupta S. Wang Y. (2009). Extracellular heat shock protein 60, cardiac myocytes, and apoptosis. *Circ. Res.* 105 1186–1195. 10.1161/CIRCRESAHA.109.209643 19875724 PMC2949276

[B35] Kobeissy F. H. Ottens A. K. Zhang Z. Liu M. C. Denslow N. D. Dave J. R. (2006). Novel differential neuroproteomics analysis of traumatic brain injury in rats. *Mol. Cell. Proteomics* 5 1887–1898. 10.1074/mcp.M600157-MCP200 16801361

[B36] Koh P. O. (2012). Melatonin attenuates decrease of protein phosphatase 2A subunit B in ischemic brain injury. *J. Pineal Res.* 52 57–61. 10.1111/j.1600-079X.2011.00918 21790776

[B37] Koh P. O. (2013). Ferulic acid attenuates the injury-induced decrease of protein phosphatase 2A subunit B in ischemic brain injury. *PLoS One* 8:e54217. 10.1371/journal.pone.0054217 23349830 PMC3547913

[B38] Lacoste B. Angeloni D. Dominguez-Lopez S. Calderoni S. Mauro A. Fraschini F. (2015). Anatomical and cellular localization of melatonin MT1 and MT2 receptors in the adult rat brain. *J. Pineal Res.* 58 397–417. 10.1111/jpi.12224 25726952

[B39] Landino L. M. Skreslet T. E. Alston J. A. (2004). Cysteine oxidation of tau and microtubule-associated protein-2 by peroxynitrite modulation of microtubule assembly kinetics by the thioredoxin reductase system. *J. Biol. Chem.* 279 35101–35105. 10.1074/jbc.M405471200 15184375

[B40] Lee E. J. Lee M. Y. Chen H. Y. Hsu Y. S. Wu T. S. Chen S. T. (2005). Melatonin attenuates gray and white matter damage in a mouse model of transient focal cerebral ischemia. *J. Pineal Res.* 38 42–52. 10.1111/j.1600-079X.2004.00173.x 15617536

[B41] Lee M. Lee S. Hong Y. (2014). Melatonin plus treadmill exercise synergistically promotes neurogenesis and reduce apoptosis in focal cerebral ischemic rats. *FASEB J.* 28:877.17.

[B42] Lee Y. C. Chuang C. Y. Lee P. K. Lee J. S. Harper R. W. Buckpitt A. B. (2008). TRX-ASK1-JNK signaling regulation of cell density-dependent cytotoxicity in cigarette smoke-exposed human bronchial epithelial cells. *Am. J. Physiol. Lung. Cell Mol. Physiol.* 294 L921–L931. 10.1152/ajplung.00250.2007 18281606

[B43] Lehnardt S. Schott E. Trimbuch T. Laubisch D. Krueger C. Wulczyn G. (2008). A vicious cycle involving release of heat shock protein 60 from injured cells and activation of toll-like receptor 4 mediates neurodegeneration in the CNS. *J. Neurosci.* 28 2320–2331. 10.1523/JNEUROSCI.4760-07.2008 18322079 PMC6671170

[B44] Li X. B. Ding M. X. Ding C. L. Li L. L. Feng J. Yu X. J. (2018). Toll-Like receptor 4 promotes the phosphorylation of CRMP2 via the activation of Rho-kinase in MCAO rats. *Mol. Med. Rep.* 18 342–348. 10.3892/mmr.2018.8968 29749502 PMC6059689

[B45] Lin L. Kim S. C. Wang Y. Gupta S. Davis B. Simon S. I. (2007). HSP60 in heart failure: abnormal distribution and role in cardiac myocyte apoptosis. *Am. J. Physiol. Heart Circ. Physiol.* 293 H2238–H2247. 10.1152/ajpheart.00740.2007 17675567

[B46] Liu F. Grundke-Iqbal I. Iqbal K. Gong C. X. (2005). Contributions of protein phosphatases PP1, PP2A, PP2B and PP5 to the regulation of tau phosphorylation. *Eur. J. Neurosci.* 22 1942–1950. 10.1111/j.1460-9568.2005.04391.x 16262633

[B47] Llanos S. Efeyan A. Monsech J. Dominguez O. Serano M. (2006). A high-throughput loss-of-function screening identifies novel p53 regulators. *Cell Cycle* 5 1880–1885. 10.4161/cc.5.16.3140 16929179

[B48] Ma O. K. Sucher N. J. (2004). Molecular interaction of NMDA receptor subunit NR3A with protein phosphatase 2A. *Neuroreport* 15 1447–1450. 10.1097/01.wnr.0000132773.41720.2d 15194871

[B49] Matsuda-Matsumoto H. Iwazaki T. Kashem M. A. Harper C. Matsumoto I. (2007). Differential protein expression profiles in the hippocampus of human alcoholics. *Neurochem. Int.* 51 370–376. 10.1016/j.neuint.2007.04.001 17513015

[B50] Mauriz J. L. Collado P. S. Veneroso C. Reiter R. J. González-Gallego J. (2013). A review of the molecular aspects of melatonin’s anti-inflammatory actions: recent insights and new perspectives. *J. Pineal Res.* 54 1–14. 10.1111/j.1600-079X.2012.01014.x 22725668

[B51] Messens J. Silver S. (2006). Arsenate reduction: thiol cascade chemistry with convergent evolution. *J. Mol. Biol.* 362 1–17. 10.1016/j.jmb.2006.07.002 16905151

[B52] Mozaffarian D. Benjamin E. J. Go A. S. Arnett D. K. Blaha M. J. Cushman M. (2015). Heart disease and stroke statistics–2015 update: a report from the American Heart Association. *Circulation* 131 e29–e322. 10.1161/CIR.0000000000000152 25520374

[B53] Okuno S. Saito A. Hayashi T. Chan P. H. (2004). The c-Jun N-terminal protein kinase signaling pathway mediates Bax activation and subsequent neuronal apoptosis through interaction with Bim after transient focal cerebral ischemia. *J. Neurosci.* 24 7879–7887. 10.1523/JNEUROSCI.1745-04.2004 15356200 PMC6729938

[B54] Park D. J. Shah F. A. Koh P. O. (2018). Quercetin attenuates neuronal cells damage in a middle cerebral artery occlusion animal model. *J. Vet. Med. Sci.* 80 676–683. 10.1292/jvms.17-0693 29563391 PMC5938200

[B55] Polcyn R. Capone M. Hossain A. Matzelle D. Banik N. L. Haque A. (2017). Neuron specific enolase is a potential target for regulating neuronal cell survival and death: implications in neurodegeneration and regeneration. *Neuroimmunol. Neuroinflamm.* 4 254–257. 10.20517/2347-8659.2017.59 29423430 PMC5800407

[B56] Quach T. T. Honnorat J. Kolattukudy P. E. Khanna R. Duchemin A. M. (2015). CRMPs: critical molecules for neurite morphogenesis and neuropsychiatric diseases. *Mol. Psychiatry* 20 1037–1045. 10.1038/mp.2015.77 26077693

[B57] Ramos E. Patiño P. Reiter R. J. Gil-Martín E. Marco-Contelles J. Parada E. (2017). Ischemic brain injury: new insights on the protective role of melatonin. *Free Radic. Biol. Med.* 104 32–53. 10.1016/j.freeradbiomed.2017.01.005 28065781

[B58] Rodriguez C. Mayo J. C. Sainz R. M. Antolin I. Herrera F. Martin V. (2004). Regulation of antioxidant enzymes: a significant role for melatonin. *J. Pineal Res.* 36 1–9. 10.1046/j.1600-079X.2003.00092.x 14675124

[B59] Sahoo M. Jena L. Rath S. N. Kumar S. (2016). Identification of suitable natural inhibitor against influenza A (H1N1) neuraminidase protein by molecular docking. *Genomics Inform.* 14 96–103. 10.5808/GI.2016.14.3.96 27729839 PMC5056903

[B60] Sahu S. Nag D. S. Swain A. Samaddar D. P. (2017). Biochemical changes in the injured brain. *World J. Biol. Chem.* 8 21–31. 10.4331/wjbc.v8.i1.21 28289516 PMC5329711

[B61] Sarnat H. B. (2013). Clinical neuropathology practice guide 5-2013: markers of neuronal maturation. *Clin. Neuropathol.* 32 340–369. 10.5414/NP300638 23883617 PMC3796735

[B62] Shah F. A. Gim S. A. Kim M. O. Koh P. O. (2014). Proteomic identification of proteins differentially expressed in response to resveratrol treatment in middle cerebral artery occlusion stroke model. *J. Vet. Med. Sci.* 76 1367–1374. 10.1292/jvms.14-0169 24998396 PMC4221170

[B63] Shah F. A. Gim S. A. Sung J. H. Jeon S. J. Kim M. O. Koh P. O. (2016). Identification of proteins regulated by curcumin in cerebral ischemia. *J. Surg. Res.* 201 141–148. 10.1016/j.jss.2015.10.025 26850195

[B64] Shah F. A. Park D. J. Gim S. A. Koh P. O. (2015). Curcumin treatment recovery the decrease of protein phosphatase 2A subunit B induced by focal cerebral ischemia in Sprague-Dawley rats. *Lab. Anim. Res.* 31 134–138. 10.5625/lar.2015.31.3.134 26472966 PMC4602080

[B65] Sontag E. Luangpirom A. Hladik C. Mudrak I. Ogris E. Speciale S. (2004). Altered expression levels of the protein phosphatase 2A are associated with Alzheimer disease pathology. *J. Neuropathol. Exp. Neurol.* 63 287–301. 10.1093/jnen/63.4.287 15099019

[B66] Stroev S. A. Gluschenko T. S. Tjulkova E. I. Spyrou G. Rybnikova E. A. Samoilov M. O. (2004). Preconditioning enhances the expression of mitochondrial antioxidant thioredoxin-2 in the forebrain of rats exposed to severe hypobaric hypoxia. *J. Neurosci. Res.* 78 563–569. 10.1002/jnr.20282 15468176

[B67] Suzuki Y. Nakagomi S. Namikawa K. Kiryu-Seo S. Inagaki N. Kaibuchi K. (2003). Collapsin response mediator protein- 2 accelerates axon regeneration of nerve-injured motor neurons of rat. *J. Neurochem.* 86 1042–1050. 10.1046/j.1471-4159.2003.01920.x 12887701

[B68] Tan D. X. Manchester L. C. Terron M. P. Flores L. J. Tamura H. Reiter R. J. (2007). Melatonin as a naturally occurring co-substrate of quinone reductase-2, the putative MT3 melatonin membrane receptor: hypothesis and significance. *J. Pineal Res.* 43 317–320. 10.1111/j.1600-079X.2007.00513.x 17910598

[B69] Truettner J. S. Hu K. Liu C. L. Dietric W. D. Hu B. (2009). Subcellular stress response and induction of molecular chaperones and folding proteins after transient global ischemia in rats. *Brain Res.* 1249 9–18. 10.1016/j.brainres.2008.10.032 18996359 PMC2670784

[B70] Valko M. Leibfritz D. Moncol J. Cronin M. T. Mazur M. Telser J. (2007). Free radicals and antioxidants in normal physiological functions and human disease. *Int. J. Biochem. Cell Biol.* 39 44–84. 10.1016/j.biocel.2006.07.001 16978905

[B71] Venegas C. Garcia J. A. Escames G. Ortiz F. Lopez A. Doerrier C. (2012). Extrapineal melatonin: analysis of its subcellular distribution and daily fluctuations. *J. Pineal Res.* 52 217–227. 10.1111/j.1600-079X.2011.00931.x 21884551

[B72] Vriend J. Reiter R. J. (2015). Melatonin feedback on clock genes: a theory involving the proteasome. *J. Pineal Res.* 58 1–11. 10.1111/jpi.12189 25369242

[B73] Wang B. Tian S. Wang J. Han F. Zhao L. Wang R. (2015). Intraperitoneal administration of thioredoxin decreases brain damage from ischemic stroke. *Brain Res.* 1615 89–97. 10.1016/j.brainres.2015.04.033 25935696

[B74] Wang Y. Chen L. Hagiwara N. Knowlton A. A. (2010). Regulation of heat shock protein 60 and 72 expression in the failing heart. *J. Mol. Cell Cardiol.* 48 360–366. 10.1016/j.yjmcc.2009.11.009 19945465 PMC2814075

[B75] Wiederstein M. Sippl M. J. (2007). ProSA-web: interactive web service for the recognition of errors in three-dimensional structures of proteins. *Nucleic Acids Res.* 35 407–410. 10.1093/nar/gkm290 17517781 PMC1933241

[B76] Wu J. X. Zhang L. Y. Chen Y. L. Yu S. S. Zhao Y. Zhao J. (2015). Curcumin pretreatment and post-treatment both improve the antioxidative ability of neurons with oxygen-glucose deprivation. *Neural Regen. Res.* 10 481–489. 10.4103/1673-5374.153700 25878600 PMC4396114

[B77] Yang W. Tiffany-Castiglioni E. Koh H. C. Son I. H. (2009). Paraquat activates the IRE1/ASK1/JNK cascade associated with apoptosis in human neuroblastoma SH-SY5Y cells. *Toxicol. Lett.* 191 203–210. 10.1016/j.toxlet.2009.08.024 19735704

[B78] Yang X. Zhang X. Li Y. Han S. Howells D. W. Li S. (2016). Conventional protein kinase Cβ-mediated phosphorylation inhibits collapsin response-mediated protein 2 proteolysis and alleviates ischemic injury in cultured cortical neurons and ischemic stroke-induced mice. *J. Neurochem.* 137 446–459. 10.1111/jnc.13538 26788931

[B79] Yin W. Signore A. P. Iwai M. Cao G. Gao Y. Chen J. (2008). Rapidly increased neuronal mitochondrial biogenesis after hypoxic-ischemic brain injury. *Stroke* 39 3057–3063. 10.1161/STROKEAHA.108.520114 18723421 PMC2726706

[B80] Yoshimura T. Kawano Y. Arimura N. Kawabata S. Kikuchi A. Kaibuchi K. (2005). GSK-3β regulates phosphorylation of CRMP-2 and neuronal polarity. *Cell* 120 137–149. 10.1016/j.cell.2004.11.012 15652488

[B81] Zhang J. N. Koch J. C. (2017). Collapsin response mediator protein-2 plays a major protective role in acute axonal degeneration. *Neural Regen. Res.* 12 692–695. 10.4103/1673-5374.206631 28616018 PMC5461599

[B82] Zhang X. Y. Chen D. C. Xiu M. H. Yang F. D. Tan Y. L. He S. (2012). Thioredoxin, a novel oxidative stress marker and cognitive performance in chronic and medicated schizophrenia versus healthy controls. *Schizophr. Res.* 143 301–306. 10.1016/j.schres.2012.11.017 23238053

[B83] Zhang Z. Ottens A. K. Sadasivan S. Kobeissy F. H. Fang T. Hayes R. L. (2007). Calpain-mediated collapsin response mediator protein-1,2, and-4 proteolysis after neurotoxic and traumatic brain injury. *J. Neurotrauma* 24 460–472. 10.1089/neu.2006.0078 17402852

[B84] Zoete V. Cuendet M. A. Grosdidier A. Michielin O. (2011). SwissParam: a fast force field generation tool for small organic molecules. *J. Comput. Chem.* 32 2359–2368. 10.1002/jcc.21816 21541964

